# A Novel Role of Ume6 in *Candida albicans* in Regulation of Oxidative Stress Tolerance

**DOI:** 10.3390/jof12050308

**Published:** 2026-04-23

**Authors:** Yanting Wang, Mengsen Zhu, Zhishang Shi, Lin Liu, Yijun Gu, Xiaoxiang Zhou, Hangqi Zhu, Jiacheng Zhao, Qilin Yu, Mingchun Li

**Affiliations:** Key Laboratory of Molecular Microbiology and Technology, Ministry of Education, Department of Microbiology, College of Life Sciences, Nankai University, No. 94 of Weijin Road, Nankai District, Tianjin 300071, China

**Keywords:** *Candida albicans*, oxidative tolerance, autophagy, apoptosis, Ume6

## Abstract

Oxidative stress is one of the major environmental stresses that the fungal pathogen *Candida albicans* frequently encounters. In view of the negative regulatory effect of Ume6 on autophagy in *Saccharomyces cerevisiae* and the close link between autophagy and oxidative stress in mammals, we explored the regulatory effect of Ume6 on autophagy and oxidative stress in *C. albicans* in this study. Here, we identify the transcriptional regulator Ume6 as a key positive regulator of autophagy under oxidative stress conditions. Deletion of *UME6* resulted in reduced autophagy levels under H_2_O_2_ treatment, correlating with reduced transcriptional expression of core autophagy-related genes. Although *UME6* deletion alone did not alter H_2_O_2_ sensitivity, it significantly exacerbated the sensitivity of a catalase mutant, revealing a functional role for Ume6 in oxidative stress tolerance. Intriguingly, we discovered that 3-methyladenine (3-MA), a canonical autophagy inhibitor in other systems, acts as an autophagy activator in *C. albicans*, promoting Atg8 transport to the vacuole and enhancing autophagy levels. This 3-MA-induced autophagy alleviated oxidative stress damage, as evidenced by improved growth and protection of vacuolar membrane integrity in H_2_O_2_-treated cells. Furthermore, deletion of *UME6* or nitrogen starvation reduced apoptosis under oxidative stress, including decreased Annexin-V binding, metacaspase activation, mitochondrial membrane depolarization, and mitochondrial cytochrome c release. This study uncovers the critical role of Ume6 in governing oxidative stress, autophagy, and apoptosis.

## 1. Introduction

*Candida albicans* is a major opportunistic fungal pathogen capable of causing severe, life-threatening systemic infections, particularly in immunocompromised individuals [1]. While it commonly resides as a commensal on mucosal surfaces, it can transition into a lethal pathogen when host defenses are breached. During infection, *C. albicans* is intensely confronted by phagocytes, such as macrophages and polymorphonuclear cells, which utilize the production of reactive oxygen species (ROS) as a primary antimicrobial burst to eliminate fungal cells [2]. Although basal levels of ROS function as vital signaling molecules, their excessive accumulation triggers oxidative stress, leading to significant cellular damage [3]. Consequently, the capacity to scavenge ROS and maintain redox homeostasis is a critical factor determining the survival and virulence of *C. albicans* within the host environment [4,5].

When *C. albicans* infects host cells, healthy hosts rely on the more established ROS-producing processes established by immune cells, such as macrophages, as an important antimicrobial defense mechanism [6]. Currently, ROS is being used as a novel antimicrobial to gradually reduce the use of conventional antimicrobials [7]. Consistent with this, *C. albicans* also has a set of mechanisms for evading oxidative damage by immune cells. The expression of antioxidant genes often plays an important role in the detoxification process, including genes encoding catalase (*CAT1*), glutathione peroxidase (*GPX*), and superoxide dismutase (*SOD*), as well as genes encoding components of the glutathione/glutaredoxin (*GSH1*, *TTR1*) and thioredoxin systems (*TSA1*, *TRX1*, *TRR1*), etc. [8]. And the direct activation of signaling pathways will also contribute to resistance to oxidative stress. Three classical signaling pathways in *C. albicans* are the Cap1 transcription factor, the Hog1 stress-activated protein kinase, and the Rad53 DNA damage checkpoint kinase [9,10,11]. In addition, studies have shown that ROS can be used as an inducer of autophagy during nutrient deprivation, and cells can degrade damaged mitochondria through the autophagy pathway to avoid oxidative damage [12,13].

Macroautophagy/autophagy is a highly conserved evolutionary process from yeast to mammalian cells. In this process, excess proteins and damaged organelles in the cytoplasm are transported to vacuoles or lysosomes for degradation [14]. Under normal physiological conditions, eukaryotic cells maintain a basal level of autophagy but upregulate the autophagy for cell adaptation and survival in response to external stimuli, such as oxidative stress, nutrient deficiency, and other stress conditions [15]. For instance, in granulosa cells and osteoblasts, ROS-induced autophagy has been shown to promote damage clearance and inhibit apoptosis through key signaling pathways, including AMPK and mTOR [16,17]. Given its important role in cell homeostasis, the dysregulation of autophagy is closely linked to numerous pathologies [18]. Furthermore, in *Saccharomyces cerevisiae*, Gtr1p functions as a critical regulator coordinating the cellular response to oxidative stress by regulating autophagic activity [19]. To date, almost 40 autophagy-related genes (*ATGs*) have been identified by genetic screens for autophagy-defective mutants in yeast, serving to help the cell maintain optimal autophagic levels for homeostasis [20]. Similarly, autophagy plays a critical role in *Candida albicans* as an indispensable defense mechanism for maintaining virulence and responding to host-derived environmental challenges. It has been reported that Protein Phosphatase 2A (PP2A) affects drug resistance and biofilm formation by participating in the phosphorylation of Atg proteins [21]. Additionally, *C. albicans* utilizes Ccz1-mediated mitophagy to selectively clear damaged mitochondria, thereby effectively reducing the accumulation of reactive oxygen species (ROS) and preventing irreversible cellular damage [22]. These findings uncover the tight internal connection between cell homeostasis, stress signaling, and autophagic regulation. Nevertheless, the detailed mechanisms of autophagy in *C. albicans* and the precise link between autophagy and oxidative stress tolerance remain to be fully elucidated.

Previous studies have shown that Ume6 was identified in *C. albicans* as a transcriptional regulator that is particularly important in hyphal filament extension in response to a variety of hyphal filament-inducing conditions. In vitro and in vivo experiments have shown that *ume6*Δ/Δ can form germ tubes but is significantly defective for filamentation and is attenuated for virulence [23]. In *Saccharomyces cerevisiae*, Ume6 was identified as a negative regulator of *ATG8* transcription to regulate autophagy activity [24]. Despite the well-documented regulatory effect of Ume6 on autophagy in *Saccharomyces cerevisiae*, its specific role in the fungal pathogen *Candida albicans*, particularly under oxidative stress, remains poorly understood. Here, we explored the regulatory role of Ume6 in the degradation of autophagy marker proteins and the transcriptional expression of core autophagy-related genes. Additionally, we examined the impact of *UME6* deficiency on cellular tolerance to oxidative stress. Finally, we investigated the role of Ume6-mediated autophagy in oxidative stress-induced apoptosis. Collectively, this study aims to determine whether Ume6 functions as a transcriptional regulator of autophagy in *Candida albicans* under H_2_O_2_ treatment conditions.

## 2. Materials and Methods

### 2.1. Growth Conditions

The *C. albicans* BWP17 strain (genotype details in Table S1) was employed as the parental strain due to its multiple auxotrophic markers, which facilitate genetic manipulation. To compensate for its uridine auxotrophy and ensure optimal physiological growth, the yeast extract-peptone-dextrose (YPD) medium (1% yeast extract, 2% glucose, 2% peptone) was supplemented with 80 μg/mL uridine for all routine cultures. The medium used for selective culture was synthetic complete (SC) medium (0.67% yeast nitrogen base without amino acids, 2% glucose, 0.2% corresponding amino acid mixture). SC medium containing 100 mg/mL 5-fluoroorotic acid (5-FOA) was used for selecting *URA*-free strains. Nitrogen starvation (SD-N) medium (0.052% KCl, 0.152% KH_2_PO_4_, 0.052% MgSO_4_, 1% glucose, 0.1% 1000× trace elements solution, 0.1% 1000× vitamin solution) was used to achieve starvation conditions, and H_2_O_2_ was added to achieve oxidative stress conditions. The strains were incubated on a shaker at 160 r/min and 30 °C until the logarithmic growth phase before performing each of the following experiments.

### 2.2. Strains

The *C. albicans* strains, plasmids, and primers used in this study are listed in Tables S1 and S2, respectively. All deletion strains in this experiment were obtained with the wild-type strain BWP17 background and produced by PCR-mediated two-step homologous recombination. The detailed procedures are described in Supplemental Information.

### 2.3. Oxidative Stress Susceptibility Test

The oxidative stress conditions involved in this experiment were hydrogen peroxide (H_2_O_2_). We observed the growth of different strains in solid and liquid media, respectively. The oxidative stress reagent was added to the YPD solid medium at the corresponding concentration. The optical density at 600 nm (OD_600_) of each strain during the logarithmic growth phase was adjusted to 0.2. The cells were then spotted on solid medium using 2 μL 10-fold serial dilutions. After incubation at 30 °C for about 48 h, we observed the growth difference between different strains. To test the effect of deletion of *UME6* on the growth of *C. albicans* in liquid medium, the fungal cells were suspended in YPD medium to an OD_600_ of 0.1, and then cultured at 30 °C with shaking for 4 h. H_2_O_2_ at a final concentration of 2 mM was added to the cultures. The cells were further cultured at 30 °C with shaking. The OD_600_ of *C. albicans* was measured every 4 h by a UV-visible spectrophotometer (BioRad, Hercules, CA, USA).

### 2.4. RNA Extraction and Real-Time Quantitative PCR (RT-qPCR)

To determine the expression of relevant genes under oxidative stress conditions, the strain during the logarithmic growth phase continued to be cultured in YPD medium containing 4 mM H_2_O_2_ for 30 min. Total RNA was extracted using an EastepTM Total RNA Extraction Kit (Promega, Madison, WI, USA). The total RNA was used for reverse transcriptional synthesis of cDNA with an oligo (dT)-primered RT reagent Kit (Promega, Madison, WI, USA). The SYBR Green qPCR SuperMix (TransGen Biotech, Beijing, China) was used for RT-PCR analysis. *ACT1* transcripts were used as an endogenous control for the quantitative PCR. All samples were taken in triplicate, independent experiments.

### 2.5. Vacuolar Membrane Permeabilization (VMP) Detection

*C. albicans* cultured to the logarithmic growth phase were treated with the appropriate stress, and the cells were collected and washed twice with YPD liquid medium. The resuspension was incubated for 30 min at 5 μg/mL C-DCFDA, protected from light at 30 °C with shaking. 5-(6)-carboxy-2,7-dichlorofluorescein diacetate (C-DCFDA, Sigma-Aldrich, St. Louis, MO, USA) is a membrane-permeable probe that is hydrolyzed by intracellular esterases into its fluorescent form, C-DCF. In healthy cells, C-DCF is actively sequestered and retained within the vacuoles, and its leakage into the cytosol indicates a loss of vacuolar membrane integrity. The ratio of the number of cells filled with green fluorescence to the total number of cells was counted by fluorescence microscopy to reflect the vacuolar membrane permeability (VMP). The higher the ratio, the more permeable the vesicle membrane is.

### 2.6. Fluorescence Microscopy

The distribution of GFP-Atg8 protein inside/outside the vesicles under oxidative stress or other conditions was observed to reflect autophagy. The strains treated with different conditions were collected and washed twice with PBS buffer. The resuspension was incubated with FM4-64 (2.5 μg/mL; Sigma-Aldrich, St. Louis, MO, USA) for 30 min in the dark at 30 °C to label the vacuolar membranes. After staining, the cells were washed twice with PBS to remove excess dye. The intracellular localization of GFP-Atg8 relative to the vacuoles was then observed using fluorescence microscopy (Olympus, Tokyo, Japan). Cells containing GFP-Atg8 were observed using green fluorescence filters, whereas cells stained with FM4-64 were observed using TRITC/Texas Red filters.

### 2.7. Protein Extraction and Western Blotting

Different treated cells containing the GFP-Atg8 fusion protein were collected. Mixed cells with glass beads and shaken well on a vortex mixer to produce cell extracts. The protein expression level was detected by SDS-PAGE and Western blotting. GFP monoclonal antibody (1:3000; MBL, Nagoya, Japan) and Tubulin monoclonal antibody (1:3000; Novus Biologicals, Centennial, CO, USA) were used as primary antibodies to detect GFP and alpha-Tubulin (as a loading control), respectively. HRP-conjugated goat anti-mouse IgG (1:5000; BioRad, Hercules, CA, USA) was used as the secondary antibody.

### 2.8. ROS Generation in the Cytosol and Mitochondria

Intracellular and mitochondrial reactive oxygen species (ROS) accumulation was assessed using the Reactive Oxygen Species Assay Kit (S0033S, Beyotime, Shanghai, China) and MitoSOX^TM^ Red (M36007, Thermo Fisher, Waltham, MA, USA). *C. albicans* cells (5 × 10^6^ cells/mL) were incubated in the presence of H_2_O_2_ (4 mM, 4 h) or SD-N (2 h) + H_2_O_2_ (4 mM, 4 h) at 30 °C. The cells were then suspended in PBS. DCFH-DA (10 µM) and MitoSOX^TM^ Red (1 µM) were added to the suspension. After incubation at 30 °C for 30 min, the cells were washed, and the fluorescent cells were detected by the Guava^®^easyCyte™ flow cytometer. The intracellular ROS was analyzed by excitation at 488 nm and emission at 525/30 nm, while the mitochondrial ROS was analyzed by excitation at 405 nm and emission at 583/26 nm.

### 2.9. Apoptosis Detection

Apoptosis of *C. albicans* in the presence of H_2_O_2_ (4 mM, 4 h) or SD-N (2 h) + H_2_O_2_ (4 mM, 4 h) was detected by Guava Nexin^®^ Assay. Cells were collected by centrifugation and resuspended in 100 μL PBS (about 1 × 10^6^ cells/sample). Add 100 μL Guava Nexin Reagent to each sample and incubate for 20 min at 30 °C in the dark. The Guava Nexin^®^ Assay utilizes Annexin V-PE (excitation wavelength 488 nm, emission wavelength 583/26 nm) and the cell impermeant dye 7-AAD (excitation wavelength 488 nm, emission wavelength 695/50 nm) to detect phosphatidylserine (PS) on the external membrane and cell membrane structural integrity of cells so that the cells could be distinguished into non-apoptotic, early apoptotic, late stage apoptotic and dead cells by flow cytometer.

### 2.10. Detection of Mitochondrial Membrane Potential (ΔΨm)

Mitochondrial membrane potential plays a key role in vital mitochondrial functions, and its dissipation is a hallmark of mitochondrial dysfunction. The fluorescent cationic dye 5,5,6,6′-tetrachloro-1,1′,3,3′-tetraethylbenzimi-dazoylcarbocyanine iodide (JC-1) (40705ES03, Yeasen, Shanghai, China) can be used to examine changes in MMP. Different treated (4 mM H_2_O_2_ 4 h or SD-N 2 h + 4 mM H_2_O_2_ 4 h) cells were collected, washed in PBS, and incubated with 1 μg/mL JC-1 at 30 °C for 30 min. At high mitochondrial membrane potential, JC-1 forms J-aggregates (excitation wavelength 488 nm, emission wavelength 695/50 nm), whereas at low mitochondrial membrane potential, JC-1 remains in its monomer state (excitation wavelength 488 nm, emission wavelength 525/30 nm).

### 2.11. Detection of Metacaspase Activity

CaspACE™ FITC-VAD-FMK In Situ Marker is a fluorescent analog of the pan caspase inhibitor carbobenzoxy-valyl-alanyl-aspartyl-[O-methyl]-fluoromethylketone (Z-VAD-FMK), which can detect activation of metacaspases. 5 × 10^6^ different treated (4 mM H_2_O_2_ 4 h or SD-N 2 h + 4 mM H_2_O_2_ 4 h) *C. albicans* cells were collected and washed in PBS. Add 200 μL staining solution containing CaspACE™ FITC-VAD-FMK In Situ Marker (G7461, Promega, Madison, WI, USA) to the cells at a final concentration of 10 µM. After incubation for 1 h at 30 °C, stained cells could be detected by a flow cytometer with excitation and emission settings of 488 nm and 525/30 nm.

### 2.12. Release of Cytochrome c

Mitochondria play key roles in apoptosis, a central step being the release of cytochrome c (cyt c) across the outer mitochondrial membrane into the cytoplasm [25]. *C. albicans* cells during the logarithmic growth phase were cultured and treated in 200 mL medium at 30 °C, collected, and washed twice with PBS. Cells were pretreated with TE buffer (0.05 M EDTA, 0.1 M Tris-HCl, 2.5% mercaptoethanol, pH 8.5) for 10 min, and then *C. albicans* spheroplasts were generated from snailase-treated (1.2 M sorbitol, 0.02 M trisodium citrate, 0.1 M EDTA-2Na, 0.02 M Na_2_HPO_4_, 2% snailase, 15 min) cells. Spheroplasts were washed once with 1.2 M sorbitol and resuspended in 500 μL mitochondrial buffer (210 mM sucrose, 70 mM Mannitol, 1 mM EDTA, 1 mM EGTA, 1.5 mM MgCl_2_, 10 mM Hepes, pH 7.2). The 500 μL cell suspension was homogenized with a homogenizer and then centrifuged at 200× *g* for 1 min. The supernatant was collected and subjected to 13,780× *g* for 15 min. The supernatant was collected again to estimate the levels of cytosolic cytochrome c, and the pellet was resuspended in mitochondrial buffer to estimate the levels of mitochondrial cytochrome c. Protein quantification was carried out using the BCA protein kit according to the manufacturer’s instructions (PC0020, Solarbio, Beijing, China). Mouse polyclonal anti cytochrome c (ab13575, Abcam, Cambridge, UK) and m-IgG^κ^ BP-HRP (sc-516102, Santa Cruz Biotechnology, Dallas, TX, USA) were used for primary and secondary antibodies, respectively.

## 3. Results

### 3.1. Ume6 Positively Regulates the Autophagy Activation Process Under H_2_O_2_ Treatment

**Figure 1:**
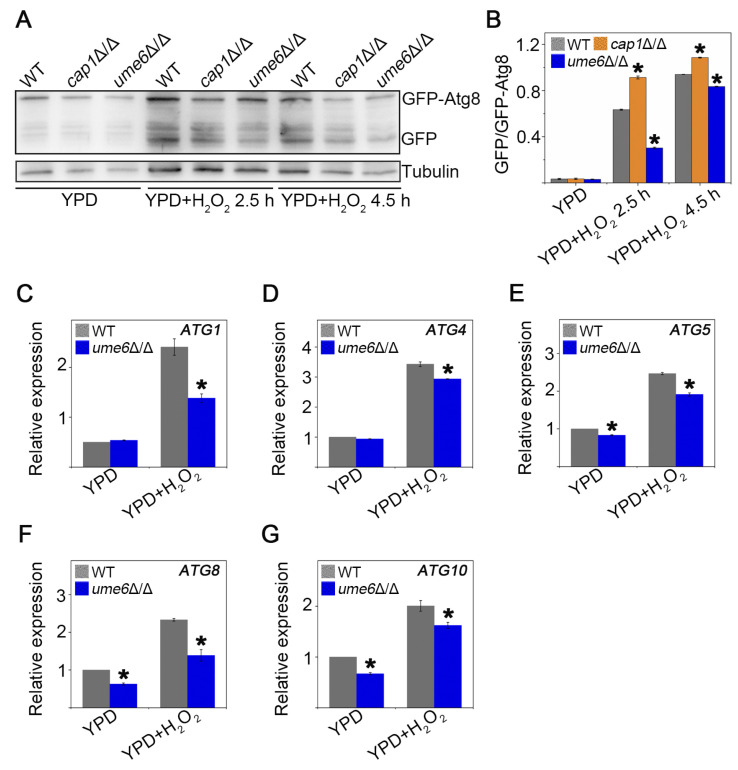
Ume6 positively regulates the autophagy activation process under H_2_O_2_ conditions. (**A**) Western blotting of GFP-Atg8 and GFP in the tested strains. The fungal cells cultured in YPD or YPD containing 4 mM H_2_O_2_ were lysed, and then the cellular total proteins were isolated. GFP-Atg8 and GFP of each sample were detected by Western blotting using the anti-GFP antibody. Tubulin was used as a loading control. (**B**) The GFP-Atg8 signals and the single GFP signals were quantified using Fiji software (version 1.54f, ImageJ), and the percentage of single GFP signals against the GFP-Atg8 signals was shown. (**C**–**G**) Cells in logarithmic growth phase were cultured in YPD medium with 4 mM H_2_O_2_ for further 30 min and harvested for RNA extraction and cDNA preparation to analyze the expression of *ATG1*, *ATG4*, *ATG5*, *ATG8*, and *ATG10*. Data were given as the mean ± SE of three experiments. * *p* < 0.05.

Previous studies have shown that Ume6 acts along with a histone deacetylase complex, including Sin3 and Rpd3, to lead to an increase in Atg8 and a concomitant increase in autophagic activity in response to nitrogen starvation in *Saccharomyces cerevisiae* [24]. To investigate whether Ume6 regulates autophagy in *C. albicans*, we used GFP-Atg8 as a marker, whose degradation to GFP is an indicator of autophagy activation [26]. *CAP1* encodes a basic region-leucine zipper (bZip) transcriptional regulatory protein that can regulate the expression of oxidative stress-induced genes [27,28]. After treatment with H_2_O_2_, the cleavage of GFP from GFP-Atg8 in the *cap1*Δ/Δ strain, which was subjected to more intense oxidative stress, was increased compared with the WT strain, whereas the autophagic flux of the *ume6*Δ/Δ strain decreased, suggesting that deletion of *UME6* inhibits the autophagy-activating process of the cells under H_2_O_2_ conditions (Figure 1A and quantified in Figure 1B). We also observed the degradation of Atg8 in *ume6*Δ/Δ under nitrogen starvation conditions and found that the deletion of *UME6* also resulted in the inhibition of autophagy activation, which suggested that Ume6 may respond to H_2_O_2_ and nitrogen starvation by a similar mechanism (Figure S1). Then RT-qPCR was performed to evaluate the effect of *UME6* deletion on the expression of some core genes related to autophagy (Figure 1C–G). The results showed a noticeable decrease in the expression of *ATG1*, *ATG4*, *ATG5*, *ATG8*, and *ATG10* in *ume6*Δ/Δ as compared to WT under H_2_O_2_ treatment, suggesting that Ume6 regulates autophagy by controlling the transcriptional level of *ATG* genes. Taken together, Ume6 positively regulates the autophagy flux under H_2_O_2_ conditions.

### 3.2. Deletion of UME6 Increases the Oxidative Stress Sensitivity of cat1Δ/Δ Strains in C. albicans

**Figure 2:**
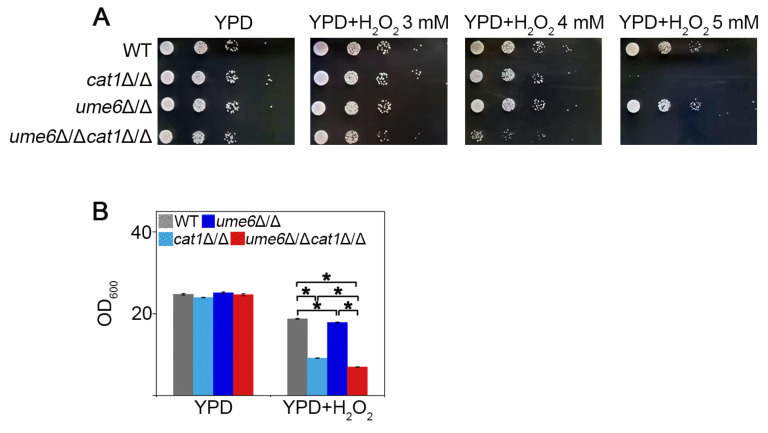
Deletion of *UME6* increases the oxidative stress sensitivity of *cat1*Δ/Δ strains in *C. albicans*. (**A**) Growth of each strain on the YPD solid plates with or without different concentrations of H_2_O_2_. (**B**) OD_600_ of each strain, which was measured after being cultured in YPD containing 2 mM H_2_O_2_ for 20 h. * indicates significant differences between the mutants and the WT strain, * *p* < 0.05.

Oxidative stress can induce antioxidant reactions and autophagy, thereby alleviating oxidative damage to biomolecules and organelles [29]. To investigate the function of Ume6 in oxidative stress response, we tested the growth of the WT, *cap1*Δ/Δ, and *ume6*Δ/Δ under H_2_O_2_ treatment (Figure S2). The results indicated that *cap1*Δ/Δ showed sensitivity to H_2_O_2_ while *ume6*Δ/Δ showed no significant difference compared to WT. Given that some oxidative stress signaling modules and antioxidant systems are still functioning to resist ROS, including catalase, the glutathione system, the thioredoxin system and mitogen-activated protein kinase (MAPK) pathways [30,31,32], it is speculated that the presence of some important oxidative stress response genes would alleviate the damage caused by H_2_O_2_, which might make the regulatory role of Ume6 on the oxidative stress response process obscured. Therefore, we attempted to construct the *ume6*Δ/Δ*cat1*Δ/Δ double mutant after failing to obtain the *ume6*Δ/Δ*cap1*Δ/Δ double-deficient strain. As expected, the growth of *ume6*Δ/Δ*cat1*Δ/Δ was obviously weakened compared to *cat1*Δ/Δ in the presence of H_2_O_2_ (Figure 2A). Moreover, in the YPD liquid medium containing H_2_O_2_, we found that the growth of both *ume6*Δ/Δ and *ume6*Δ/Δ*cat1*Δ/Δ showed inhibition compared to WT and *cat1*Δ/Δ (Figure 2B). Together, this suggests Ume6 is a significant part of the oxidative stress reaction.

### 3.3. 3-Methyladenine Acts as an Autophagy Activator in C. albicans

**Figure 3:**
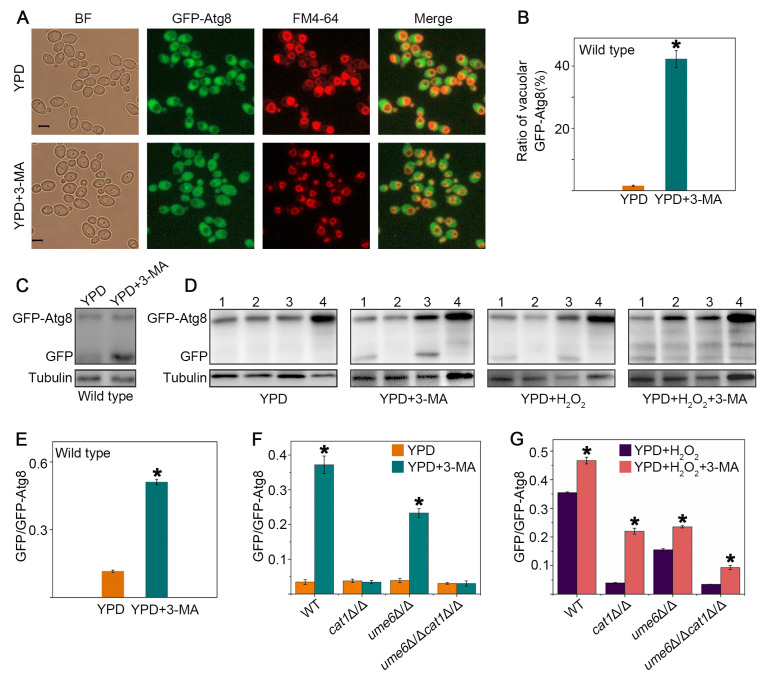
3-MA acts as an autophagy activator in *C. albicans*. (**A**) The localization of GFP-Atg8 (green) under different culture conditions was observed using a fluorescence microscope. Vacuoles were stained with FM4-64 (red). Bar = 5 μm. (**B**) The percent of cells with vacuolar-accumulated GFP-Atg8 in (**A**), 2 h after treatment with 5 mM 3-MA. * *p* < 0.05. (**C**–**G**) The degradation of GFP-Atg8 in the WT strain and other strains with deleted treated with the stress shown was evaluated by Western blotting and was quantified using Fiji software (version 1.54f, ImageJ). Strains 1 to 4 are WT, *ume6*Δ/Δ, *cat1*Δ/Δ, and *ume6*Δ/Δ*cat1*Δ/Δ, respectively. The concentration of 3-MA is 5 mM, and the concentration of H_2_O_2_ is 4 mM. Total cell lysates under different treatment conditions were prepared and detected by anti-GFP antibody and anti-tubulin antibody. Tubulin was used as a loading control. * *p* < 0.05.

As an important selective inhibitor of phosphatidylinositol-3-kinase (PI3K), 3-methyladenine (3-MA) can block the formation of autophagic vesicles in animals [33]. The role of 3-MA in autophagy regulation in fungi remains unknown. Figure 3A revealed that GFP-Atg8 in the WT strain cultured in YPD liquid medium containing 3-MA was gradually transported from the cytoplasm to the vacuole, while most of the GFP-Atg8 in the WT strain cultured in YPD liquid medium remained in the cytoplasm, indicating that the transport of Atg8 was activated under 3-MA treatment. To determine the percent of cells with vacuolar-accumulated GFP-Atg8, the number of cells with vacuolar-accumulated GFP-Atg8 and the total number of cells in each field were counted. The percentage of cells with vacuolar-accumulated GFP-Atg8 was then calculated as the number of cells with vacuolar-accumulated GFP-Atg8 divided by the total number of cells (Figure 3B). The results show that the percentage of cells with vacuolar-accumulated GFP-Atg8 increased in the WT strain after treatment with 3-MA. And the cleavage of GFP from GFP-Atg8 significantly increased due to 3-MA when examined by Western blotting analysis (Figure 3C and quantified in Figure 3E). Likewise, we further validated the function of 3-MA by analyzing the cleavage of GFP from GFP-Atg8 in WT, *ume6*Δ/Δ, *cat1*Δ/Δ, and *ume6*Δ/Δ*cat1*Δ/Δ strains under the multiple treatment conditions illustrated in Figure 3D (quantified in Figure 3F,G). We found that autophagic activity in *cat1*Δ/Δ and *ume6*Δ/Δ*cat1*Δ/Δ was almost blocked under H_2_O_2_ treatment compared to the WT strain, whereas autophagy was activated in these strains after treatment with 3-MA. Notably, regardless of the treatment conditions, GFP-Atg8 was much more expressed in the *ume6*Δ/Δ*cat1*Δ/Δ strain than in other strains, possibly because unidentified pathways affect the expression or degradation of Atg8 in the double-mutant strain, a phenomenon that warrants further investigation. Taken together, 3-MA can act as an autophagy activator in *C. albicans* under certain conditions.

### 3.4. The 3-MA Treatment Alleviates Cellular Damage from Oxidative Stress

**Figure 4:**
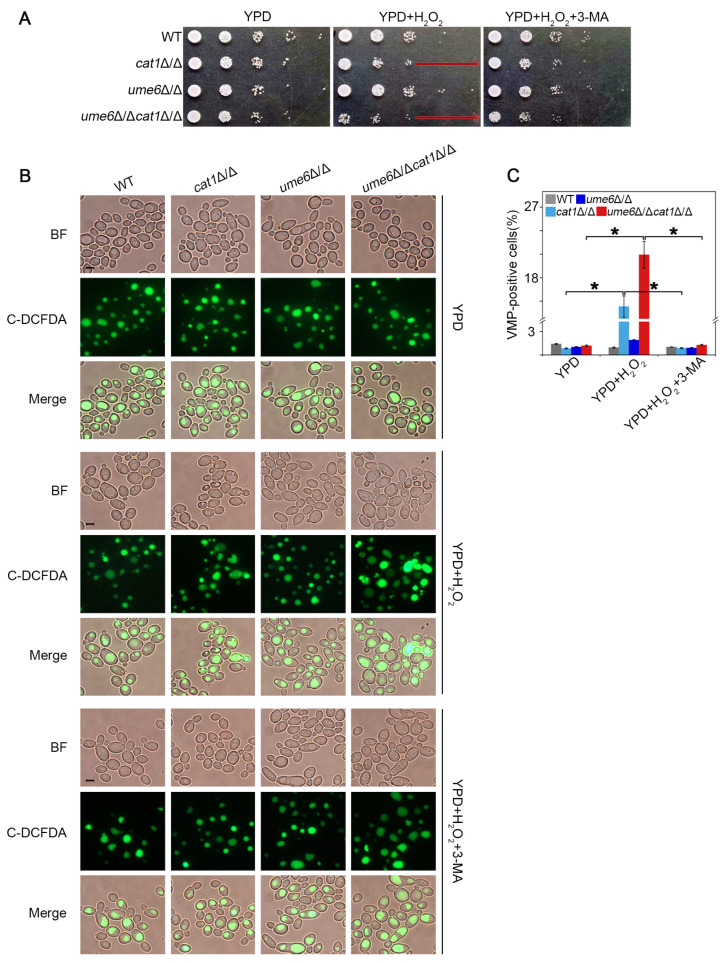
The 3-MA treatment alleviates cellular damage from oxidative stress. (**A**) Effect of 3-MA addition on the growth of the strains on the plates containing H_2_O_2_. Arrows indicate the enhanced growth density of *cat1*Δ/Δ and *ume6*Δ/Δ*cat1*Δ/Δ strains in the presence of 3-MA compared to the untreated controls. (**B**) Cells were cultured in YPD and YPD containing 4 mM H_2_O_2_ with or without 3-MA treatment for 1 h. The treated cells were stained with C-DCFDA (green) and observed by fluorescence microscopy. Bar = 5 μm. (**C**) Statistical analysis of VMP-positive cells. At least 10 fields were observed for analysis. * indicates significantly statistical difference among treatments, *p* < 0.05.

Given the finding that Ume6 has an effect on both oxidative stress sensitivity and autophagic flux under H_2_O_2_ treatment (Figure 1 and Figure 2), we were interested in discovering more links between autophagy and oxidative stress. Therefore, we analyzed the growth of each strain on the plates containing 4 mM H_2_O_2_ and the plates containing 4 mM H_2_O_2_ and 5 mM 3-MA. The *cat1*Δ/Δ and *ume6*Δ/Δ*cat1*Δ/Δ strains with defective oxidative stress responses due to deletion of the *CAT1* gene exhibited strong oxidative stress sensitivity on solid plates containing H_2_O_2_, whereas the survival stress of the cells was well relieved by the addition of 3-MA (Figure 4A).

The Vacuolar Membrane Permeability (VMP) is one of the important indicators involved in oxidative stress-related cell death in yeast cells [34]. We detected VMP of the *C. albicans* cells by C-DCFDA, a cell-permeant esterase substrate that can serve as a viability probe [35]. In the absence of H_2_O_2_, both WT and the mutants had regular FDA fluorescence in the vacuoles. Under the treatment with H_2_O_2_ for 1 h, *cat1*Δ/Δ and *ume6*Δ/Δ*cat1*Δ/Δ had obvious whole-cell FDA distribution, whereas the VMP of these strains cultured in the presence of 3-MA was not significantly different from the VMP of the normal condition (Figure 4B). Likewise, the presence of 3-MA was still able to protect the integrity of the vacuolar membrane after 2 h of H_2_O_2_ treatment (Figure S3), indicating that the vacuolar membrane damage caused by oxidative stress can be alleviated by 3-MA. Taken together, these data showed that the autophagy induced by 3-MA plays an important role in the response to oxidative stress in *C. albicans*.

### 3.5. The Autophagy Activation Process Induced by Ume6 or Nitrogen Starvation Helps Cells to Survive in H_2_O_2_ Treatment

**Figure 5:**
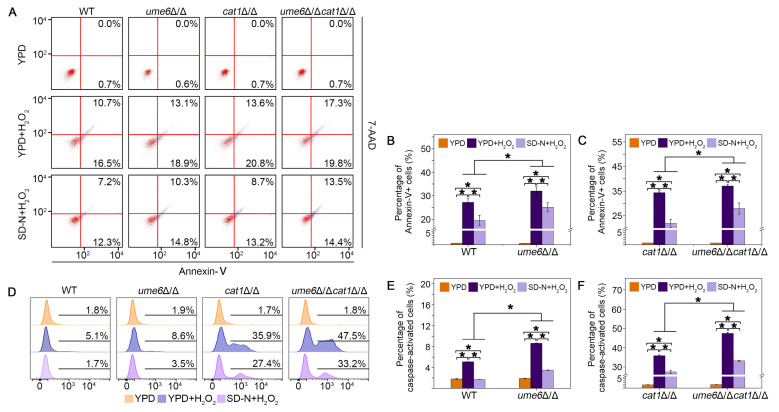
Nitrogen starvation-induced autophagy helps the *C. albicans* cells to survive in H_2_O_2_ treatment. The cells were cultured in YPD and YPD containing 4 mM H_2_O_2_ (4 h) with or without SD-N pretreatment (2 h). (**A**) non-apoptotic (red, **lower left** quadrant, Annexin V-negative, 7-AAD-negative), early apoptotic (blue, **lower right** quadrant, Annexin V-positive, 7-AAD-negative), late apoptotic and necrotic cells (blue, **upper right** quadrant, Annexin V-positive and 7-AAD-positive) can be differentiated. (**B**,**C**) Quantification of Annexin-positive cells induction observed in (**A**). * *p* < 0.05. (**D**) Metacaspase activity was assessed by FITC-VAD-FMK assay. The percentage of stained cells was shown in the histogram in (**E**,**F**). * *p* < 0.05.

To confirm that this is the role of autophagy in alleviating cellular damage caused by oxidative stress, we introduced the nitrogen starvation treatment (SD-N), which induces autophagy [36]. Based on the fact that apoptosis can be induced by oxidative stress [37], we measured several apoptosis markers in H_2_O_2_-treated *C. albicans* cells with or without the pretreatment of SD-N. We first used Annexin-V/7-AAD double-staining to differentiate apoptotic and necrotic cells. Annexin-V detects PS externalization in early apoptotic cells. PS exists in the inner leaflet of the plasma membrane, but when apoptosis occurs, it can be exposed to the outer leaflet, and Annexin V combines with PS. As a fluorescent DNA dye that binds poly(dG-dC) regions, 7-AAD can be used as a dead cell discriminating dye due to its exclusion from live cells with intact membranes [38]. WT, *ume6*Δ/Δ, *cat1*Δ/Δ, and *ume6*Δ/Δ*cat1*Δ/Δ strains were stained with Annexin-V/7-AAD after H_2_O_2_ treatment with or without SD-N pretreatment, and the proportions of early apoptotic cells (lower right) and late apoptotic cells (upper right) to the total number of cells were examined by flow cytometry and are presented in Figure 5A. Cells in which the autophagy program was activated by SD-N pretreatment were better able to survive in H_2_O_2,_ and the proportion of Annexin-V positive cells, including the apoptotic and necrotic cells, was reduced compared to cells without SD-N pretreatment. Deletion of *UME6* resulted in a reduced level of cellular autophagy under H_2_O_2_ conditions (Figure 1), leading to an increase in the percentage of Annexin-V positive cells in the *ume6*Δ/Δ and *ume6*Δ/Δ*cat1*Δ/Δ strains compared to the WT and *cat1*Δ/Δ strains (Figure 5A–C), which is consistent with the results in Figure 2 where deletion of *UME6* resulted in an increased oxidative stress sensitivity of the cells. We further assessed the activation of metacaspases by the FITC-labeled caspase inhibitor VAD-FMK. It binds specifically to the active center of metazoan caspases, which enables a flow cytometric determination of cells with active caspases [39]. The proportion of cells with active meta-caspases was reduced after SD-N pretreatment compared to cells directly exposed to H_2_O_2_ treatment and was increased after deletion of *UME6* compared to WT and *cat1*Δ/Δ strains (Figure 5D–F). Taken together, these data support the idea that the proper activation of autophagy can help *C. albicans* cells survive better under oxidative stress.

### 3.6. Autophagy Activation by Ume6 or Nitrogen Starvation Helps C. albicans Maintain Mitochondrial Homeostasis Under H_2_O_2_ Treatment

**Figure 6:**
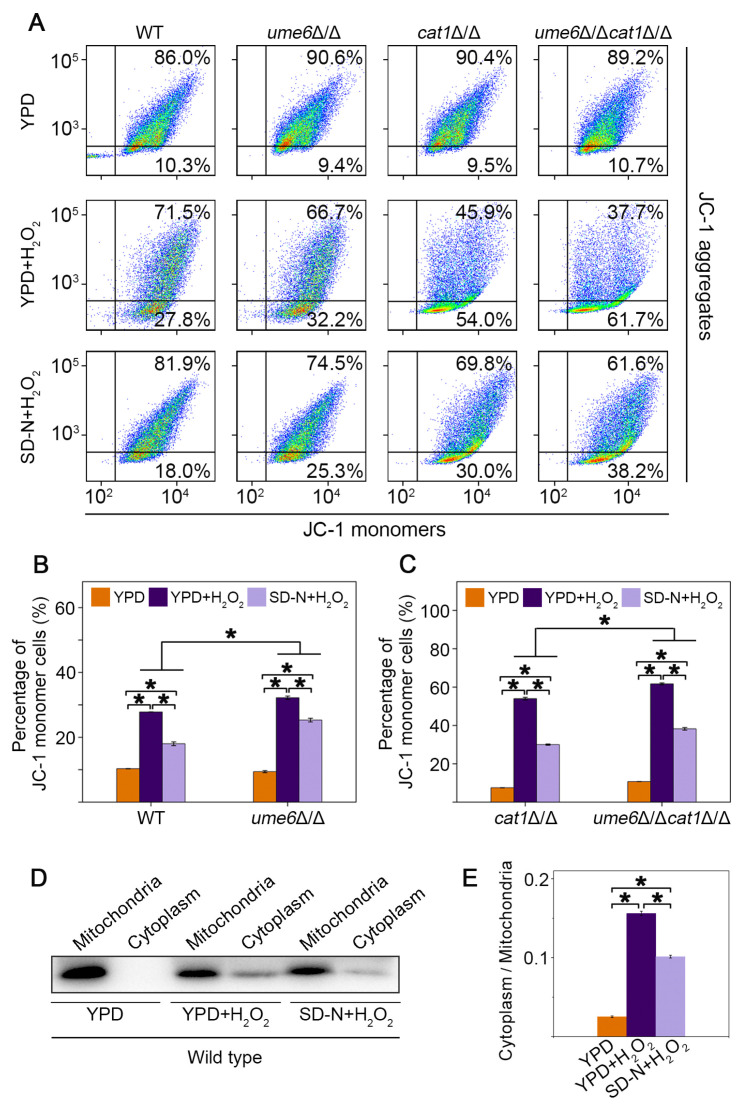
Nitrogen starvation activates autophagy and helps *C. albicans* maintain mitochondrial homeostasis under H_2_O_2_ treatment. The cells were cultured in YPD and YPD containing 4 mM H_2_O_2_ (4 h) with or without SD-N pretreatment (2 h). (**A**) Mitochondrial membrane potential was evaluated using JC-1 staining. Representative flow cytometry dot plots were shown for each strain under various treatment conditions. (**B**,**C**) Statistical analysis of the percentage of cells with JC-1 monomers. Mitochondrial and cytosolic cytochrome c levels were shown by Western blotting (**D**), and the results of Fiji analysis (version 1.54f, ImageJ) were shown in (**E**). The asterisk (*) indicates significantly statistical difference among the treatments, *p* < 0.05.

JC-1 dye was used as a mitochondrial membrane potential indicator to assess mitochondrial membrane depolarization. In the mitochondrial matrix of normal cells, the cationic JC-1 dye produces a red fluorescence in the form of J-aggregates. In contrast, in apoptotic cells with changes in mitochondrial membrane potential, JC-1 is released in the cytoplasm and produces a green fluorescence [40]. A decrease in the mean fluorescence intensity of J-aggregates would indicate the release of JC-1 dye from the mitochondria, in accordance with its depolarization. The percentage of JC-1 monomers for WT, *ume6*Δ/Δ, *cat1*Δ/Δ, and *ume6*Δ/Δ*cat1*Δ/Δ was 27.8%, 32.2%, 54.0% and 61.7%, respectively, under the treatment of H_2_O_2_. And the percentage of JC-1 monomers decreased to 18.0%, 25.3%, 30.0% and 38.2% for these strains facing oxidative stress after nitrogen starvation treatment, indicating that pretreatment with nitrogen starvation alleviated the mitochondrial depolarization of the cells in the face of oxidative stress. Also, the percentage of JC-1 monomers decreased in *ume6*Δ/Δ and *ume6*Δ/Δ*cat1*Δ/Δ strains compared to the WT and *cat1*Δ/Δ strain (Figure 6A,B). In combination with Figure 1, these results suggest that Ume6-mediated autophagy has an important role in the maintenance of cellular mitochondrial membrane potential under H_2_O_2_ treatment. Release of pro-apoptotic factors such as cytochrome c from mitochondria to the cytoplasm has been considered “the point of no return” of apoptosis [41]. To further evaluate the release of cytochrome c, the untreated and treated cells were subjected to mitochondria fractionation. Mitochondrial cytochrome c content was assessed by Western blotting analysis (Figure 6D). Compared to YPD culture conditions, the WT strain exhibited a higher degree of cytochrome c release from mitochondria after H_2_O_2_ treatment. The quantification of band intensity suggested that after nitrogen starvation pretreatment, the release of mitochondrial cytochrome c from the wild-type strain was reduced under oxidative stress (Figure 6E). Taken together, the autophagy activation induced by Ume6 or SD-N helps the fungal cells maintain mitochondrial homeostasis under H_2_O_2_ treatment.

## 4. Discussion

The fungicidal mechanism employed by the first line of defense of our body, the innate immune system, involves the production of reactive oxygen species (ROS) like superoxide, hydrogen peroxide, etc., which can lead to irreversible damage and apoptosis of fungal cells. Some previous studies also suggested that amphotericin B and fluconazole activity involves the production of endogenous ROS induced by oxidative damage [42]. Consequently, there is much interest in the strategies employed by *C. albicans* to evade the oxidative killing by macrophages and neutrophils. Our understanding of how *C. albicans* senses and responds to ROS has significantly increased in recent years. A well-characterized response of eukaryotic microbes to ROS is the rapid induction of mRNAs that encode oxidative stress detoxification and repair proteins. Interestingly, *C. albicans* is considerably more resistant to oxidative stress than the benign model yeasts, *Schizosaccharomyces pombe* and *Saccharomyces cerevisiae* [2]. However, the basis for this resistance does not appear to be due to differences in transcriptional responses to oxidative stress, as all three fungi appear to induce a similar set of core antioxidant genes following exposure to H_2_O_2_ [43,44]. Therefore, this study is expected to further enrich the oxidative stress tolerance pathway in *C. albicans*.

Autophagy is a very important catabolic pathway in cells that can adapt to changes in physiological stresses and nutrient availability. High levels of ROS can activate autophagy in mammal cells, while autophagy can eliminate cell damage caused by ROS [44,45]. Autophagy in yeast cells can be induced under stress stimulation or nutrient-deprived conditions [46], but the relationship between autophagy and oxidative stress remains to be deeply explored. In this study, we found that deletion of *UME6* inhibited the activation of autophagy under oxidative stress by repressing the expression of autophagy-related genes (Figure 1). Furthermore, the deletion of *UME6* in the *cat1*Δ/Δ background significantly enhanced cellular sensitivity to oxidative stress (Figure 2). Compared to the WT strain, *cat1*Δ/Δ strains suffer from exacerbated oxidative stress due to the absence of catalase [32]. Under such compromised stress-response conditions, the proper induction of autophagy becomes especially vital for cell survival. We speculate that the *ume6*Δ/Δ*cat1*Δ/Δ strain is unable to initiate an adequate level of autophagy due to the absence of *UME6*; consequently, the cells fail to effectively alleviate severe oxidative damage, leading to a marked reduction in oxidative stress tolerance. Therefore, we further investigated whether and how autophagy affects oxidative stress response. The results showed that 3-MA can act as an autophagy activator in *C. albicans* under certain conditions (Figure 3), and the presence of 3-MA was able to protect the integrity of the vacuolar membrane under H_2_O_2_ treatment to alleviate the oxidative stress to which the cells were subjected (Figure 4). And in Figure 5, cells activated with the autophagy program by SD-N pretreatment were able to survive better under H_2_O_2_ treatment whereas the proportion of Annexin V-positive cells, including apoptotic and necrotic cells, increased in strains with reduced autophagy levels due to deletion of *UME6*, which is consistent with the results in Figure 2 where deletion of *UME6* resulted in an increased oxidative stress sensitivity of the *cat1*Δ/Δ strain. The above results suggest that proper activation of autophagy may play an important role in the survival of cells under oxidative stress and imply an important link between autophagy, oxidative stress, and apoptosis.

Apoptosis is a form of programmed cell death, required to eliminate dead and unnecessary cells and to regulate cell homeostasis. In addition to multicellular organisms, unicellular organisms, such as yeast, are also able to undergo programmed cell death that exhibits many hallmarks of apoptosis, including PS externalization, DNA fragmentation, metacaspase activation, ROS accumulation, loss of mitochondrial membrane potential, and elevations in cytosolic and mitochondrial Ca^2+^ [47]. Oxidative damage from excessive ROS generation has been demonstrated to damage the cells severely, inducing apoptosis and cell cycle arrest [48]. Further examination of apoptosis-related markers showed that strains with reduced levels of autophagy due to deletion of *UME6* exhibited higher levels of apoptosis under H_2_O_2_ conditions, such as elevated metacaspase activity, reduced mitochondrial membrane potential, and increased transfer of cytochrome c from the mitochondria to the cytoplasm, whereas the proper activation of autophagy under oxidative stress conditions reduced the apoptosis rate and maintains the mitochondrial homeostasis of cells (Figure 5 and Figure 6).

Although the mitochondria represent the principal source of ROS required for autophagy induction, we did not observe the effect of autophagy on ROS levels during oxidative stress in Figure S4, which suggests that the activated autophagy can relieve the stress of cell survival under oxidative stress by directly affecting the level of apoptosis in *C. albicans*. Our study demonstrated that Ume6 regulates the autophagy-dependent oxidative stress tolerance pathway, revealing an important role of Ume6 in regulating the oxidative stress tolerance and establishing an important link between autophagy, apoptosis, and oxidative stress in *C*. *albicans*, which provides additional theoretical basis for how *C*. *albicans* responds to oxidative stress.

In conclusion, this study identifies Ume6 as a positive regulator of autophagy under oxidative stress conditions in *C. albicans*, demonstrating that Ume6 enhances autophagy level by modulating the transcription of core autophagy-related genes. Analysis of cell growth under H_2_O_2_ treatment showed that strains with deletion of the *UME6* gene were more sensitive to oxidative stress. Furthermore, our findings indicate that the activation of autophagy, whether mediated by Ume6, nitrogen starvation, or 3-MA treatment, plays a cytoprotective role against oxidative stress. These results establish a critical link between Ume6-mediated autophagy, oxidative stress tolerance, and apoptosis regulation, deepening the understanding of how *C. albicans* withstands host immune oxidative attacks and providing potential targets for antifungal therapeutic strategies.

## Data Availability

The original contributions presented in this study are included in the article/Supplementary Material. Further inquiries can be directed to the corresponding authors.

## Supplementary Materials

The following supporting information can be downloaded at: https://www.mdpi.com/article/10.3390/jof12050308/s1. Figure S1: Ume6 positively regulates the autophagy activation process under SD-N conditions. Cells cultured in YPD or SD-N medium were lysed, then cellular total proteins were isolated. The cleavage of GFP from GFP-Atg8 of each sample were detected by using the anti-GFP antibody. Tubulin was used as a loading control. Figure S2: Effect of deletion of UME6 on cell growth under oxidative stress. Growth of each strain on the YPD solid plate with or without different concentrations of H2O2 was tested. Figure S3: Cells were cultured in YPD and YPD containing 4 mM H2O2 with or without 3-MA treatment for 2 h. The treated cells were stained by C-DCFDA and observed by fluorescence microscopy. Bar = 5 μm. Figure S4: Cells cultured in the conditions shown were detected by flow cytometer to analyze the accumulation of ROS and mitochondrial ROS. Table S1: Strain and plasmid used in this study; Table S2: Primers used in this study.
