# Supplementary material for: A Novel Role of Ume6 in Candida albicans in Regulation of Oxidative Stress Tolerance
Source: J Fungi (Basel). 2026 Apr 23;12(5):308. doi: 10.3390/jof12050308 (PMC13208243; doi:10.3390/jof12050308)
Supplement: Supplementary file 1 [file jof-12-00308-s001.zip › jof-4257251-supplementary.pdf]

A novel role of Ume6 in *Candida albicans* in regulation of oxidative stress tolerance

Yanting Wang, Mengsen Zhu, Zhishang Shi, Lin Liu, Yijun Gu, Xiaoxiang Zhou, Hangqi Zhu, Jiacheng Zhao, Qilin Yu*, Mingchun Li*

Key Laboratory of Molecular Microbiology and Technology, Ministry of Education, Department of Microbiology, College of Life Sciences, Nankai University, No. 94 of Weijin Road, Nankai District, Tianjin 300071, China

* Correspondence: yuqilin@mail.nankai.edu.cn (Q.Y.); nklimingchun@163.com (M.L.)

Strains and plasmids construction

Based on the genotype of BWP17, the gene deletion strains could be screened and identified by Arginine, Histidine and Uracil. Given that *C. albicans* is a diploid organism, we choose pRS-ArgΔ*SpeI* (Amp^R *ARG4*) and pDDB57 (Amp^R *URA3*) plasmids to produce a homozygous mutant that was morphologically identical to the parental wild-type strain. Taking *ume6*Δ/Δ as an example, the Arg cassette containing the *UME6* homologous fragment was first amplified using the UME6-DR primer with pRS-ArgΔ*SpeI* as the template. The Arg cassette was transformed into WT, and *ume6Δ/UME6* was obtained after screening and validating the transformants. pDDB57 was also amplified to obtain the Ura cassette and transformed into *ume6Δ/UME6* to obtain *ume6*Δ/Δ. *ume6*Δ/Δ strains were then lined on 5-FOA plates and screened to obtain *ume6*Δ/Δ-*URA3* strains without the *URA3* fragment (referred to as *ume6*Δ/Δ in this study) in order to proceed to knockdown *CAT1* with Ura markers**.**

In the construction of related strains containing the GFP-Atg8 fusion protein, the plasmid pAU34m-GFP-Atg8 was first linearized using the endonuclease Bgl II. Then the digested product was transformed into WT and other strains.

Tables

Table S1 Strain and plasmid used in this study

**Strain / plasmid**	**Genotype and description**	**Source**
**Strains（*C. albicans*）**		
WT(BWP17)	*ura3Δ::λimm434/ura3Δ::λimm434 his1::hisG/his1::hisG arg4::hisG/arg4::hisG*	[20]
*cap1*Δ/Δ-*URA3*	*ura3Δ::λimm434/ura3Δ::λimm434 his1::hisG/his1::hisG arg4::hisG/arg4::hisG cap1::ARG4/cap1::dpl200*	This study
*cat1*Δ/Δ-*URA3*	*ura3Δ::λimm434/ura3Δ::λimm434 his1::hisG/his1::hisG arg4::hisG/arg4::hisG cat1::ARG4/cat1::dpl200*	This study
*ume6*Δ/Δ-*URA3*	*ura3Δ::λimm434/ura3Δ::λimm434 his1::hisG/his1::hisG arg4::hisG/arg4::hisG ume6::ARG4/ume6::dpl200*	This study
*ume6*Δ/Δ*cat1*Δ/Δ-*URA3*	*ura3Δ::λimm434/ura3Δ::λimm434 his1::hisG/his1::hisG arg4::hisG/arg4::hisG ume6::ARG4/ume6::dpl200 cat1:: dpl200/ cat1:: dpl200*	This study

WT-GFP-Atg8	*ura3Δ::λimm434/ura3Δ::λimm434 his1::hisG/his1::hisG arg4::hisG/arg4::hisG ATG8/GFP-ATG8::URA3*	This study
*cat1*Δ/Δ-GFP-Atg8	*ura3Δ::λimm434/ura3Δ::λimm434 his1::hisG/his1::hisG arg4::hisG/arg4::hisG cat1::ARG4/cat1::dpl200 ATG8/GFP-ATG8::URA3*	This study
*ume6*Δ/Δ-GFP-Atg8	*ura3Δ::λimm434/ura3Δ::λimm434 his1::hisG/his1::hisG arg4::hisG/arg4::hisG ume6::ARG4/ume6::dpl200 ATG8/GFP-ATG8::URA3*	This study
*ume6*Δ/Δ*cat1*Δ/Δ-GFP-Atg8	*ura3Δ::λimm434/ura3Δ::λimm434 his1::hisG/his1::hisG arg4::hisG/arg4::hisG ume6::ARG4/ume6::dpl200 cat1:: dpl200/ cat1:: dpl200 ATG8/GFP-ATG8::URA3*	This study
**Plasmids**		
pRS-*ARG4*Δ*Spe*I	Amp^R, containing *ARG4* marker	[20]
pDDB57	Amp^R, containing *URA3* marker	[20]
pAU34m-GFP-Atg8	Amp^R, containing *URA3* marker	[20]

Table S2 Primers used in this study

**Name of primer**	**Sequence (5′-3′)**	**Application**
UME6-5DR<br>UME6-3DR	TCACTCCAAACATCAATATCCTCAATTTACT TCCAATTAGTTTTATTTTTTAATCCACTGTTT CCCAGTCACGACGTT<br>TCTTTCTAATAATTATAACAAAATCATTATC ATCAAAAAAACACCACCACCAACGGCAACT GGAATTGTGAGCGGATA	For deletion of *UME6*
UME6-5det<br>UME6-3det	GCTTTGCTTTACATAATTGG<br>ATGGACCAAAATATTACGAG	
UME6-5inner<br>UME6-3inner	CGATCAACAAGCTATCTACA<br>ATTGTGACGTACGTGTGATG	

	TTTTTTTATTGTTGTCTCCCCGCTAAATTTCT	
	ATTTCAATTCAATCTCTCTCCTTTGTAGTTTC	
CAT1-5DR	CCAGTCACGACGTT	
CAT1-3DR	AGTATAGTTGGTACCCAATCTGTGTCTGTGA	
	GTATCAGCATAGGAGAACAATCTTGATTGT	
	GGAATTGTGAGCGGATA	
		For deletion of *CAT1*
CAT1-5DET	GTAGAAGAAGTTATACCGAGGAAGTTTATC	
CAT1-3DET	GGATATCTCTTCAACACTTTCCACAATTCT	
CAT1-5inner	ACGGTCAACCAATTCCAGAA	
CAT1-3inner	GGGTATTTCTTGTGTGGCCA	
	TAATAATGCTGTAGTTCCATTCATAAGGCTG	
	GAGATGGATTGCTTAAAGAACATTCAAGAT	
CAP1-5DR	TTCCCAGTCACGACGTT	
CAP1-3DR	GGGGAATTATTTGTTACTGAAGTATTTGCTT	
	TAGAACCTGAACGTTTAAATTTGGGCACTT	
	GGAATTGTGAGCGGATA	
		For deletion of *CAP1*
CAP1-5DET	ATACGATCCTACAATTGCTA	
CAP1-3DET	AATAATGAAATTGGATCGGC	
CAP1-5inner	CCGAACCTAAATCAAAGAGA	
CAP1-3inner	TGGTTCCACATGCTTCATTC	
URA3-5inner	CGCGGGATTTGGATGGTAT	For selection of strains
URA3-3inner	TCTTGGCTCTTGGTTGGTG	without the *URA* gene
ATG1-5RT	AGGCAGATTTGTGGTCGGT	For RT-qPCR
ATG1-3RT	GTAGTGGGTGATGGGCTTCT	
ATG4-5RT	TACCGGTGCAACAAGATTCA	For RT-qPCR
ATG4-3RT	ACCAGCAGCAGTTGATTTCC	
ATG5-5RT	GACTTGGGTTGCTGGACGAT	For RT-qPCR
ATG5-3RT	GCATTATTGGTTTGGCAGTGC	
ATG8-5RT	CAGAGGTTCAAGGATAGAGT	For RT-qPCR
ATG8-3RT	GTCAATTGCTAGTTTCTCGC	
ATG10-5RT	GTTGGCAGTTGGGATTTGTC	For RT-qPCR
ATG10-3RT	TATAGCCCAAACCAGCAACA	
ACT1-5RT	GGTAGACCAAGACATCAAGG	For RT-qPCR
ACT1-3RT	CCGTGTTCAATTGGGTATCT	

Supplemental figures

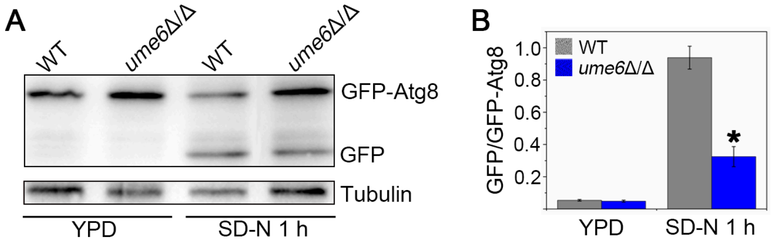

Figure S1 Ume6 positively regulates the autophagy activation process under SD-N conditions. Cells cultured in YPD or SD-N medium were lysed, then cellular total proteins were isolated. The cleavage of GFP from GFP-Atg8 of each sample were detected by using the anti-GFP antibody. Tubulin was used as a loading control.

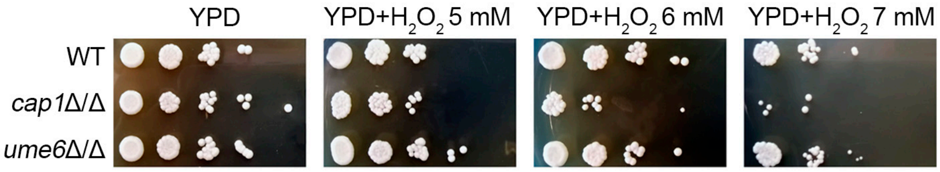

Figure S2 Effect of deletion of *UME6* on cell growth under oxidative stress. Growth of each strain on the YPD solid plate with or without different concentrations of H_2O_2 was tested.

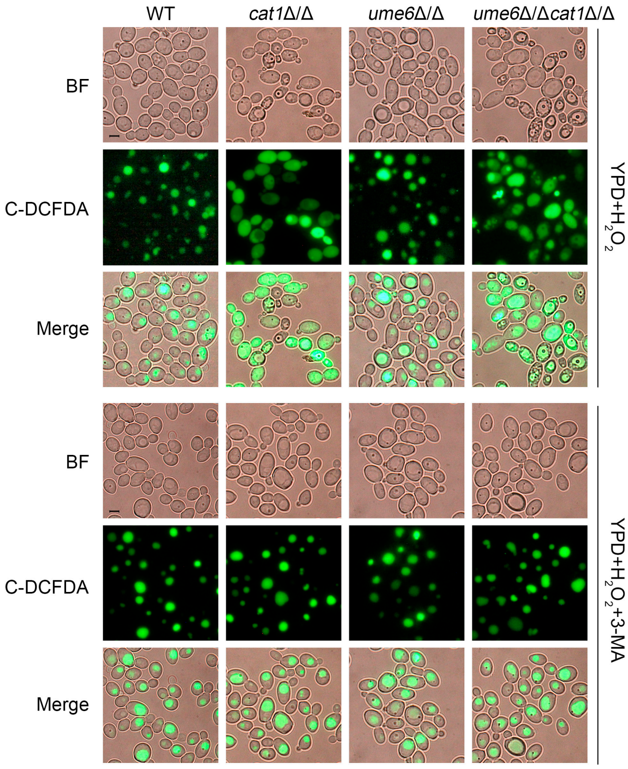

Figure S3 Cells were cultured in YPD and YPD containing 4 mM H_2O_2 with or without 3-MA treatment for 2 h. The treated cells were stained by C-DCFDA and observed by fluorescence microscopy.

Bar = 5 μm.

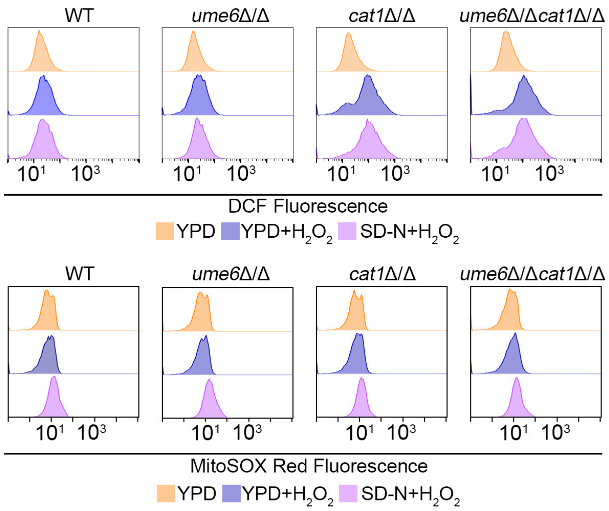

Figure S4 Cells cultured in the conditions shown were detected by flow cytometer to analyze the accumulation of ROS and mitochondrial ROS.
